# Rapamycin-loaded chitosan functionalized poly (lactic-co-glycolic acid) (PLGA) nanoparticles for potential wet age-related macular degeneration treatment: an in vivo study

**DOI:** 10.1007/s10856-026-07060-z

**Published:** 2026-07-25

**Authors:** Yuan Hu, Lijuan Wei, Haijun Yang, Jianhong Sun, Donglei Li, Qian Liu

**Affiliations:** 1 Department of Ocular Diseases, Nanchang Bright Eye Hospital, Nanchang, China; 2 Department of Ophthalmology, Hainan General Hospital (Hainan General Hospital (Hainan Affiliated Hospital of Hainan Medical University), Haikou, China; 3 Department of Ophthalmology, Baoding No.2 Central Hospital, Baoding, China

## Abstract

Wet age-related macular degeneration (Wet AMD) is a major cause of vision loss and has been linked to abnormal choroidal neovascularization (CNV). Current therapies require frequent intravitreal injections, which leads to poor patient adherence. Herein, this study was designed to investigate rapamycin-loaded chitosan-coated poly(lactic-co-glycolic acid) nanoparticles, which represent a sustained-release drug delivery system for wet AMD. The Rap@CS/PLGA NPs were synthesized using emulsion solvent evaporation, followed by coating with chitosan. The obtained Rap@CS/PLGA NPs exhibited a size of 265.9 ± 6.30 nm and encapsulation efficiency (EE) of 60.30 ± 1.20%. The drug release profile showed a biphasic pattern with reduced initial burst release (6 ± 0.6%) followed by sustained release (46 ± 4.1%) over 72 h, which indicates improved delivery control. In vitro cytotoxicity on ARPE and HUVEC cells showed minimal toxicity, with more than 85% viability at dosages up to 100 μg/mL. In vivo anti-angiogenic assays using the chick chorioallantoic membrane (CAM) model showed that the Rap@CS/PLGA group (13 ± 3 vessels) significantly reduced the vessel formation vs Rap (26 ± 2 vessels), confirming superior anti-angiogenic effects. Moreover, the laser-induced CNV mouse model, Rap@CS/PLGA NPs, significantly reduced levels of inflammatory cytokine (TNF-α and IL-6) and restored antioxidant enzyme (SOD, CAT). This showed that protection against oxidative stress in CNV-affected tissue promotes angiogenic gene expression (VEGFA, CCL2), while preserving retinal structure. These findings propose that Rap@CS/PLGA NPs can effectively and control drug delivery for wet-AMD with long-lasting efficacy.

**Graphical Abstract:**

**Figure:**
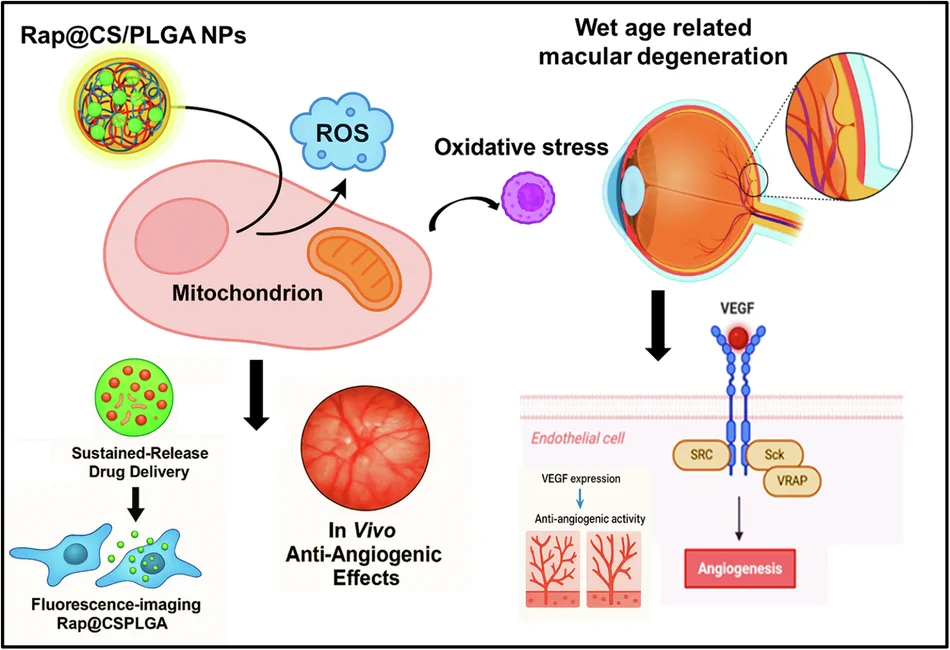

## Introduction

Age-related macular degeneration (AMD) is a predominant cause of central vision impairment among the elderly, despite the continuous rise in global life expectancy [1]. This is a degenerative disorder, and it mainly affects the macula, which is the central part of the retina that ultimately leads to irreversible impairment of vision. The advent of anti-vascular endothelial growth factor medications such as aflibercept, pegaptanib, bevacizumab, and ranibizumab has greatly changed the management of AMD. VEGF is a very important factor that plays a role in causing AMD by causing abnormal neovascularization. Although these drugs represent significant clinical milestones, they have a limited therapeutic range and precise dosing to avoid adverse effects. Thus, patients often require intravitreal injections, increasing the risk of complications such as endophthalmitis and retinal detachment [2]. Also, VEGF protects nerve cells in the retina, which, if long-term inhibition occurs, could unintentionally exacerbate retinal cell degeneration. It is also well known that not all patients respond effectively to anti-VEGF therapy [3, 4].

In the context, inflammation and oxidative stress are significant influences in the progression of AMD; thus, therapies targeting pathways that are particularly antioxidant and anti-inflammatory agents have shown potential in mitigating the disease [5]. Notably, Rap, a macrolide molecule derived from Streptomyces hygroscopicus, is gaining prominence. The FDA has approved it for use in organ transplantation, coronary restenosis, and renal cancer. Mechanistically, immunosuppression occurs with the use of sirolimus due to the inhibition of the mTOR-mechanistic target of Rap, which constrains the T-cell proliferation and activation. The SAVE study [6] evidenced that ocular application has been effective for individuals with diabetic macular edema and non-infectious uveitis. Rap has also been proven to be more effective at inhibiting the endothelial cell sprouting than VEGF alone [7]. Conversely, monotherapy with Rap for wet AMD has shown morphological improvements, yet remain a concern about its ocular side effects and limited effectiveness with prolonged use [8].

Rap’s therapeutic limits are that physicochemical properties, such as its low water solubility (2.6 μg/mL), high hydrophobicity, pH insensitivity (due to the lack of ionizable groups between pH 2-10), and susceptibility to hydrolysis and oxidation. It also displays dose-dependent toxicity and rapid systemic clearance, all of which hinder effective delivery via oral/intravenous routes in ocular applications [9]. Consequently, an effective delivery system is vital to ensure localized, sustained drug release while maintaining protective efficacy and patient comfort. To overcome these challenges, it is suggested that potential solutions can be found through the incorporation of nanotechnology-based delivery strategies in the near future [10]. Nanocarriers can improve drug targeting, internalization, and retention to increase their overall bioavailability [11, 12].

Of these, polymeric nanoparticles have gained considerable attention due to their favorable characteristics, including increased stability, prolonged release, and ease of dispersion in biological fluids [13, 14]. PLGA (poly(lactic-co-glycolic acid)) is a well-known and FDA-approved polymer which generally used for drug encapsulation due to its safety, biodegradability, and compatibility with numerous bioactive agents, including Rap [15]. However, the hydrophobic and negatively charged characteristics of PLGA can influence drug delivery in such a way that the uptake of the cells involved is reduced, as well as the release kinetics and the inflammatory response or rapid clearance of the drugs [16, 17].

Surface modification of PLGA nanoparticles is one of the approaches to deal with these barriers. To that, adding the functional materials like chitosan (CS), a natural polycationic polysaccharide derived from crustacean shells, can improve surface hydrophilicity, cellular adhesion, and drug release profiles [18]. CS coated nanoparticles have demonstrated greater mucoadhesion and solubility, which makes them promising carriers for ocular drug delivery [19, 20].

As yet, the use of chitosan-functionalized PLGA nanoparticles to deliver Rap, especially for wet AMD treatment, has not been fully studied. Herein, this study was designed to create a dual-polymer nanoparticle system to improve the ocular delivery of Rap. We hypothesize that chitosan coating enhances the permeability, cellular uptake, and overall bioavailability of PLGA-encapsulated Rap. The synthesized Rap@CS/PLGA NPs were physicochemically characterized for particle size, surface charge, drug loading efficiency, release behavior, and scleral permeability. In vitro assays, including the anti-angiogenic potential, biocompatibility, intracellular uptake, and reactive oxygen species (ROS) generation in both ARPE and HUVEC cells, were performed. In addition, the therapeutic efficacy of Rap@CS/PLGA NPs was evaluated using a laser-induced CNV mouse model, with focus on their ability to modulate inflammatory cytokines and angiogenic factors.

## Materials and methods

### Chemicals and drugs

Rapamycin (Rap) and poly(lactic-co-glycolic acid) (PLGA; 50:50) were acquired from Shanghai Yuanye Bio-Technology Co., Ltd. Polyvinyl alcohol (PVA) and chitosan (molecular weight ~120,000 Da, deacetylation degree >80%) were procured from Aladdin Reagent Co., Ltd. (Shanghai, China). Sinopharm Chemical Reagent Co., Ltd. (Beijing, China) supplied all the other analytical-grade chemicals, solvents, and reagents used.

### Synthesis of CS/PLGA NPs

PLGA nanoparticles were first prepared using the emulsion solvent evaporation technique [21]. Briefly, 50 mg of PLGA was dissolved in 2 mL of dichloromethane, referred to as the organic phase. This organic phase was then slowly added to 10 mL of 1% PVA solution under magnetic stirring. Next, probe sonication at 30% amplitude with pulse mode, 5-second on and off, was done for 2 min. This created an emulsion of PLGA nanoparticles. This mixture was then stirred for 4 h under room temperature to allow dichloromethane evaporation and PLGA nanoparticles formation. PLGA nanoparticles were then collected through centrifugation at 15,000 rpm for 20 min. They were then resuspended in water after being washed twice using deionized water.

A 0.1% (w/v) CS solution was prepared using 1% (v/v) acetic acid as a solvent, and it was filtered after preparation. The PLGA NPs were added dropwise to the CS solution with slow stirring for 30 min to enable the adsorption onto the PLGA NPs surfaces. The CS/PLGA NPs were harvested by centrifugation, and excess CS was removed by washing them with deionized water for the subsequent drug loading process.

### Preparation of Rap-loaded CS/PLGA NPs (Rap@CS/PLGA NPs)

For loading Rap into chitosan-functionalized PLGA nanoparticles, the passive incubation technique was adopted. For this, a fixed amount of Rap (5 mg) was solubilized in a small volume of ethanol, and this Rap-containing solution was slowly added to the CH-PLGA nanoparticles with gentle stirring. After that, it was allowed to incubate at room temperature for an appropriate duration of 12 h, which helped the entry of Rap into the polymeric carrier matrix. After the incubation, the Rap-loaded nanoparticles were collected, washed, and stored at 4 °C for further analysis.

#### Entrapment efficiency (%EE)

The entrapment efficiency (%EE) was determined through the centrifugation method [22]. Using ultracentrifugation at 4 °C and 15,000 rpm for 5 min, the nanoparticles were separated from the free medication. Again, centrifugation of the sample was carried out at 30 min. The collected nanoparticles were washed and then redissolved using MQ water. After sonicating the sample for 30 min, centrifugation was carried out at 5000 rpm for 15 min. Then, 100 μL of the CS/PLGA nanoparticle suspension was diluted using 5 mL of methanol. The absorbance of the diluted supernatant containing nanoparticles was measured at a wavelength of 277 nm using a UV spectrophotometer to determine the quantity of the rapamycin drug contained in the nanoparticles. The formula for calculating the EE % of the nanoparticles is: (Quantity of drug encapsulated/Quantity of drug used) x 100.

#### In vitro drug-release

The in vitro drug release investigation was undertaken with minor changes as previously reported [23]. To prepare both Rap@CS/PLGA NPs and Rap suspensions (containing 0.2% sodium carboxymethyl cellulose and 0.25% polysorbate-80), a 500 mL beaker containing phosphate-buffered saline (PBS, pH 7.4) with 0.2% (w/v) polysorbate-80 was used as the receptor medium. This solution was placed in contact with an activated cellulose dialysis membrane (molecular weight cut-off: 12–14 kDa). The system was maintained at 37 ± 0.5 °C and stirred continuously at 100 rpm using a magnetic stirrer (REMI, Mumbai, India) to ensure consistent mixing. At certain intervals, 1 mL of solution was removed and replaced with fresh dissolving media. Afterward, the Rap concentration was determined using a UV spectrophotometer (277 nm). To further elucidate the drug release mechanism, the cumulative release data were fitted to various kinetic models, including zero-order, first-order, Higuchi, and Korsmeyer–Peppas models. The correlation coefficient (R²) values were used to determine the best-fitting model. The release exponent (n) obtained from the Korsmeyer–Peppas model was used to characterize the drug release mechanism.

### Characterization of Rap@CS/PLGA NPs

To evaluate the physicochemical properties of the prepared Rap@CS/PLGA nanoparticles, a series of characterization techniques was employed. FT-IR spectroscopy was used to determine the functional groups and potential interactions between Rap, chitosan, and PLGA in both placebo and drug-loaded formulations. X-ray diffraction (XRD) analysis was used to investigate the physical state of Rap in the nanoparticles and to determine whether the drug remained crystalline or became amorphous after encapsulation. Differential scanning calorimetric studies helped understand thermal changes and the possibility of the drug and polymers being mutually compatible. On the other hand, thermogravimetric studies were employed to confirm the presence of the chitosan coating by observing the thermal degradation characteristics. Transmission electron microscopy studies were conducted to observe the morphology of the nanoparticles. Similarly, the size of the particles, the polydispersity index, and the zeta potential of the nanoparticles were measured by performing dynamic light scattering studies. Surface wettability was determined by measuring the contact angle of a water droplet on a dried film of nanoparticles, providing information on the surface’s hydrophilicity. A TAXTPlus texture analyzer, Stable Micro Systems, UK was employed to assess the adhesive behavior of PLGA and CS-coated PLGA nanoparticles. The lyophilized samples were fixed to a double-sided adhesive tape mounted on the probe. Goat scleral tissue was positioned underneath, and a 1 N force was applied for 120 seconds. Upon withdrawal of the probe, the detachment force required to separate the nanoparticles from the tissue was recorded as an indicator of their mucoadhesive strength. Finally, a stability assessment was conducted by monitoring changes in particle size and PDI over 3 and 6 months of storage at refrigerated (4 °C) and ambient (25 °C) temperatures.

### In vitro cytocompatibility on ARPE-19 and HUVEC cells

#### Cell culture

The human retinal pigment epithelial cell line (ARPE-19) was obtained from ATCC and cultured in RPMI-1640 medium supplemented with 10% fetal bovine serum (FBS) and 1% penicillin-streptomycin. Cells were maintained at 37 °C in a humidified incubator with 5% CO₂, subcultured using 0.25% trypsin-EDTA at 70–80% confluence, and used for assays between passages 5 and 10.

Human umbilical vein endothelial cells (HUVECs) were obtained from ATCC and cultured in Endothelial Cell Growth Medium-2 (EGM-2, Lonza) containing 2% FBS and endothelial growth supplements. Cells were maintained at 37 °C in a humidified incubator with 5% CO₂ and subcultured at 70–80% confluence using trypsin-EDTA. HUVECs between passages 3 and 7 were used for all experiments.

The concentrations of Rap used in the in vitro cellular assays were selected based on previously reported intravitreal and ocular cell–based rapamycin studies, and were further supported by preliminary cytocompatibility screening in ARPE-19 and HUVEC cells [24].

#### MTT assay

Cytocompatibility of nanoparticles was assessed in both ARPE-19 and HUVEC cells. Cells were seeded at 1 × 10⁴ cells/well in 96-well plates and allowed to attach for 24 h. Treatments included pure Rap, blank CS/PLGA NPs, and Rap@CS/PLGA NPs at concentrations of 20–100 μg/mL for 24 h. Triton X-100 (0.1%) served as a positive control, and serum-free media served as a negative control. After treatment, 20 μL of MTT solution (5 mg/mL in PBS) was added to each well and incubated for 4 h at 37 °C. Formazan crystals were dissolved in DMSO, and absorbance was recorded at 570 nm. Cell viability was reported as a percentage compared with untreated cells. Acridine orange staining was also performed to visualize live cells under a fluorescence microscope.

#### Reactive oxygen species (ROS) generation

Intracellular ROS levels were measured in ARPE-19 and HUVEC cells using 2′,7′-dichlorofluorescin diacetate (DCFDA). Cells were seeded in 6-well plates and treated with Rap, blank NPs, and Rap@CS/PLGA NPs (100 μg/mL) for 24 h. Following treatment, cells were incubated with 100 μM DCFDA in HBSS for 30 min at 37 °C in the dark. Cells were washed with HBSS to eliminate excess dye, followed by fluorescence observation at 488 nm excitation.

#### Cell migration (wound healing assay)

The migratory capacity of ARPE-19 and HUVEC cells was evaluated using a scratch assay. Cells were seeded in 6-well plates and grown to confluence. A sterile pipette tip was used to create a linear scratch, followed by washing with PBS. Cells were treated with pure Rap, blank NPs, or Rap@CS/PLGA NPs (100 μg/mL). Migration into the scratch area was monitored at 0 and 24 h using a phase-contrast microscope. Wound closure was quantified using ImageJ software.

#### VEGF-A assay

VEGF-A secretion was assessed in ARPE-19 and HUVEC cells using an ELISA kit (eBioscience, USA). Cells were seeded in 96-well plates (1 × 10⁴ cells/well) and treated with pure Rap, blank NPs, or Rap@CS/PLGA NPs (100 μg/mL) for 24 h. Supernatants were collected, and VEGF-A levels were determined following the manufacturer’s protocol. Absorbance was recorded at 450 nm, and VEGF-A inhibition was compared across groups.

#### Tube formation assay (HUVEC Only)

The angiogenic properties of HUVECs following treatment were assessed using a tube formation assay. Briefly, Matrigel (50 μL per well) was dispensed into a pre-cooled 96-well plate and allowed to polymerize at 37 °C for 30 min. HUVECs were then seeded onto the solidified Matrigel at a density of 2 × 10⁴ cells per well and exposed to free rapamycin, blank nanoparticles, or Rap@CS/PLGA nanoparticles (100 μg/mL) for 6–8 h. Capillary-like structures were examined using an inverted microscope, and quantitative analysis of total tube length, number of branching points, and loop formation was performed using ImageJ software equipped with the Angiogenesis Analyzer plugin.

All in vitro assays, including MTT, ROS generation, wound healing, VEGF-A secretion, and tube formation assays, were performed with at least three independent biological replicates (*n* = 3) unless otherwise stated.

### In vivo antiangiogenic assessment using the CAM assay

Fertilized chicken eggs were incubated at 37 ± 0.5 °C, 55 ± 7% relative humidity, and 50 rpm in a shaking incubator (Daihan Labtech Co. Ltd, Korea) for nine days. On the tenth day, the eggs were carefully opened at the air sac end to expose the chorioallantoic membrane (CAM). The inner shell membrane was carefully peeled away to expose the chorioallantoic membrane (CAM) and its underlying vasculature. A 0.5 mL volume of each test formulation: normal saline (negative control), 0.1 M NaOH (positive control), pure rapamycin, blank CS/PLGA nanoparticles, and Rap@CS/PLGA nanoparticles was applied directly onto the CAM surface. The samples remained in contact for 0.5, 2, and 5 min. Following treatment, the membranes were inspected for vascular reactions such as hemorrhage, hyperemia, or clotting. For the anti-angiogenic assessment, the embryos were returned to the incubator and maintained for an additional 24 h. Blood vessels within the treated CAM regions were manually counted to evaluate vascular inhibition. All CAM experiments were conducted using six independent embryos per group (*n* = 6). Vessel quantification was performed independently by two blinded observers, and the averaged counts were used for statistical analysis to reduce observer bias. Dose selection for the CAM assay followed the same rationale as the in vitro studies, based on reported ocular rapamycin literature and cytocompatibility screening [24].

### In vivo evaluation of Rap@CS/PLGA nanoparticles in a murine model of choroidal neovascularization

Healthy C57BL/6J mice (6–8 weeks old, weighing 20–30 g) were obtained from the Shanghai Laboratory Animal Center. All animal procedures complied with the ARVO guidelines and were approved by the Institutional Animal Care and Use Committee of Nanchang Bright Eye Hospital, Nanchang, China. The study was conducted in collaboration with the Department of Ophthalmology, Hainan General Hospital (Hainan Affiliated Hospital of Hainan Medical University), Haikou, China. Experimental choroidal neovascularization (CNV) was induced using a well-established laser photocoagulation method. Mice were anesthetized, and 4–5 laser burns were applied around the optic nerve head in each eye using a Micron IV image-guided laser system (Phoenix Research Laboratories). Only lesions showing cavitation bubbles, indicating rupture of Bruch’s membrane, were included. Following photocoagulation, intravitreal injections were performed using a 35-gauge needle connected to a 10 µL NanoFil syringe. Each eye received 2 μL of Rapamycin-loaded CS/PLGA nanoparticles (500 μg/mL), unloaded nanoparticles, or PBS as the control vehicle. Each experimental group consisted of six mice (*n* = 6), and both eyes were analyzed when applicable. The mice were housed in a temperature-controlled facility under a 12-h light/dark cycle and observed daily for health status. On the seventh day, animals were humanely euthanized to collect tissue samples. The eyes were carefully enucleated, fixed in 10% neutral buffered formalin, embedded in paraffin, and sectioned at a thickness of 5 µm. These sections were then stained with hematoxylin and eosin (H&E) to assess retinal structure and characterize lesion morphology. In parallel, RPE-choroid complexes were dissected for molecular and cytokine analysis. For inflammatory profiling, tissues were homogenized in RIPA buffer containing protease inhibitors, and the levels of TNF-α, IL-6, and IL-1β were quantified using commercial ELISA kits (e.g., BioLegend or R&D Systems) according to the manufacturer’s protocols. Cytokine concentrations were normalized to total protein content determined by BCA assay and expressed in pg/mg protein. To assess the antioxidant response, choroid-RPE tissue homogenates were collected from treated and control mice at day 7 post-laser induction. The activities of superoxide dismutase (SOD) and catalase (CAT) were measured using commercial assay kits (e.g., Beyotime Biotechnology, China) according to the manufacturers’ instructions. Enzyme activities were normalized against total protein content, which was determined by the BCA protein assay, and reported as units per milligram of protein (U/mg). Furthermore, total RNA was isolated from RPE-choroid complexes using TRIzol reagent (Invitrogen). Complementary DNA (cDNA) was synthesized employing the SuperScript VILO cDNA synthesis kit. Quantitative real-time PCR (qRT-PCR) was then conducted with SYBR Green master mix on an MX3005P thermal cycler (Agilent). Relative expression levels of VEGFA, CCL2, IL6, and TNF-α were normalized to GAPDH and calculated using the ΔΔCt method (Table 1). These assays provided both transcriptional and protein-level insights into the anti-inflammatory effects of Rapamycin-loaded nanoparticle treatment in the CNV model.

**Table 1:** The Primer sequences used for quantitative RT-qPCR analysis

Gene	Forward Primer (5′ → 3′)	Reverse Primer (5′ → 3′)
VEGFA	AGGGCAGAATCATCACGAAGT	AGGGTCTCGATTGGATGGCA
CCL2	CCAGCCAGATGCAATCAATG	TGGAATCCTGAACCCACTTCT
IL6	TAGTCCTTCCTACCCCAATTTCC	TTGGTCCTTAGCCACTCCTTC
TNF-α	CCCTCACACTCAGATCATCTTCT	GCTACGACGTGGGCTACAG
GAPDH	TGCACCACCAACTGCTTAGC	GGCATGGACTGTGGTCATGAG

### Statistical analysis

Data are presented as mean ± standard deviation (SD). All experiments were performed with clearly defined biological replicates, and exact sample sizes (n) are reported in the corresponding figure legends. Statistical analyses were conducted using GraphPad Prism 8.0.1. One-way ANOVA followed by Tukey’s post hoc test was applied for multiple-group comparisons. A *p*-value < 0.05 was considered statistically significant.

## Results

### Synthesis and characterization of Rap@CS/PLGA NPs

**Fig. 1:**
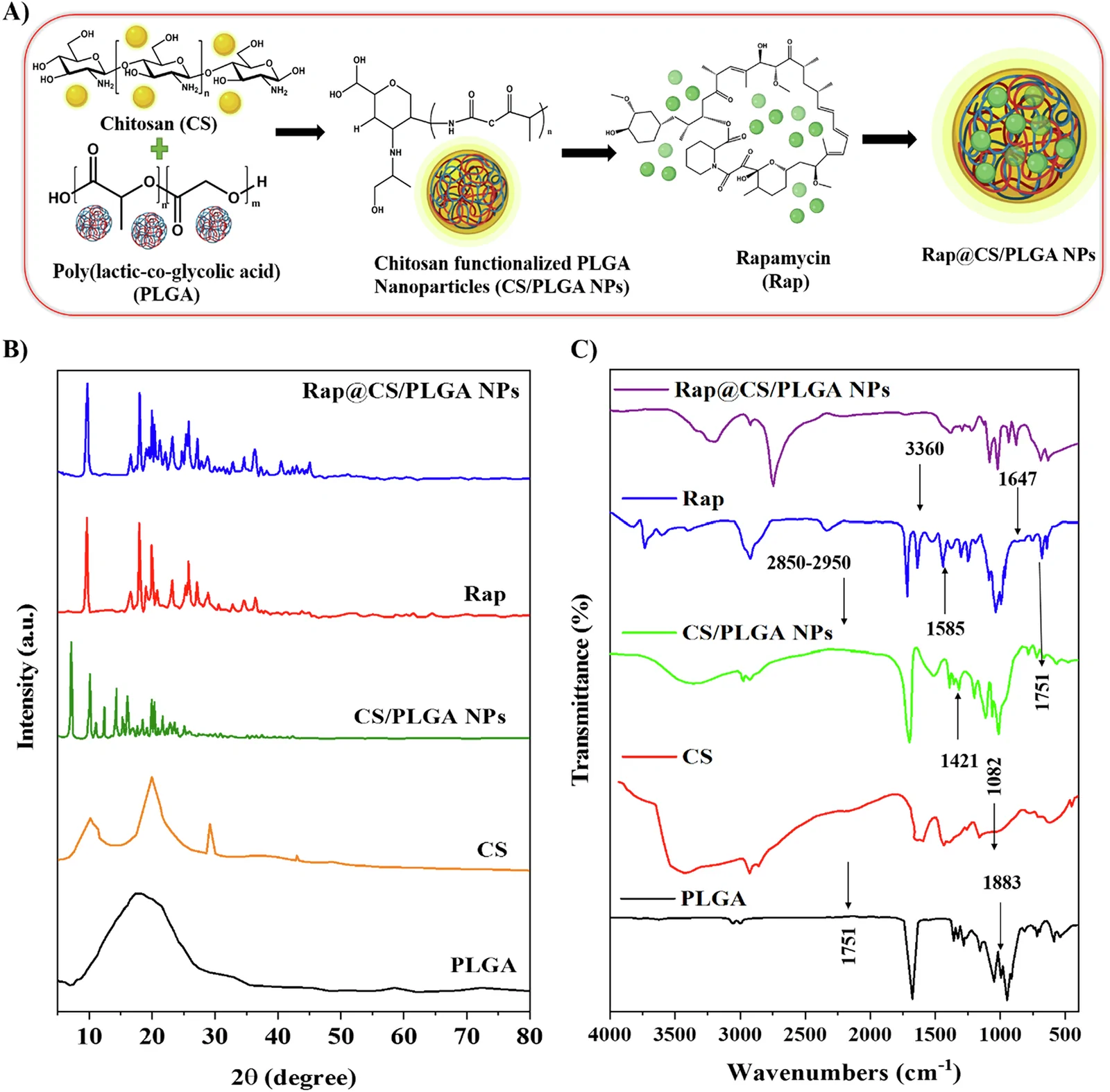
**A** The scheme illustrating that Schematic representation of the synthesis of rapamycin-loaded chitosan-functionalized PLGA nanoparticles (Rap@CS/PLGA NPs); **B** FTIR spectra of CS, PLGA, and Rap@CS/PLGA NPs display distinct absorption bands, confirming chemical interactions and successful drug encapsulation; **C** XRD profiles show the amorphous nature of the nanoparticles compared to the crystalline peaks of the raw materials, indicating reduced crystallinity after formulation

The synthesis of CS/PLGA nanoparticles was successfully achieved through an emulsion solvent evaporation technique (Fig. 1A). Following magnetic stirring, the organic phase was introduced dropwise to an aqueous phase from PLGA dissolved in DCM. DCM rapidly diffused from the organic phase to the external aqueous phase, precipitating PLGA nanoparticles. PLGA nanoparticles were mixed with chitosan to make CS/PLGA-NPs. The interactions between cationic chitosan and anionic PLGA enabled the formation of stable nanoparticles.

In physicochemical characterization, the FT-IR spectra of PLGA showed strong absorption bands at 1751 cm⁻¹ and 1082 cm⁻¹, corresponding to C=O and C–O–C stretching vibrations. Furthermore, peaks in the 2800–3000 cm⁻¹ area were attributed to CH, CH₂, and CH₃ stretching vibrations. In Chitosan, overlapping O-H and N-H stretching vibrations caused a large absorption band at 3360 cm⁻¹. Deformation bands of 1° and 2° amine groups were identified at 1647 and 1585 cm⁻¹, while C–H bending vibrations were observed between 1373 and 1421 cm⁻¹. Rap had clear FTIR peaks; however, Rap@CS/PLGA NPs had none or weaker peaks. Rap’s effective encapsulation in the polymeric matrix may obscure its functional group signals, causing this attenuation. Rap@CS/PLGA NPs exhibit slightly different peak locations compared to native PLGA and chitosan, indicating successful surface adsorption and interaction. These spectrum properties contribute to the effective formulation of the nanoparticle system, as shown in Fig. 1B.

Figure 1C displays the crystalline structure of pure Rap, which was confirmed by the XRD pattern, which showed multiple intense and sharp diffraction peaks at 7.11°, 9.79°, 10.08°, 10.99°, 12.37°, 14.08°, and 14.35°. Both Rap-loaded PLGA nanoparticles and chitosan-coated Rap-loaded PLGA nanoparticles (Rap@CS/PLGA NPs) had XRD spectra that nearly matched the diffraction patterns of their unloaded counterparts; however, these characteristic peaks were missing. When crystalline peaks vanish, Rap in the polymeric matrix changes from a crystalline to an amorphous or molecularly dispersed form. As shown in Fig. 1B, C, the results of FT-IR and XRD investigations taken together confirm that Rap was successfully encapsulated and uniformly dispersed within the nanoparticle formulation.

**Fig. 2:**
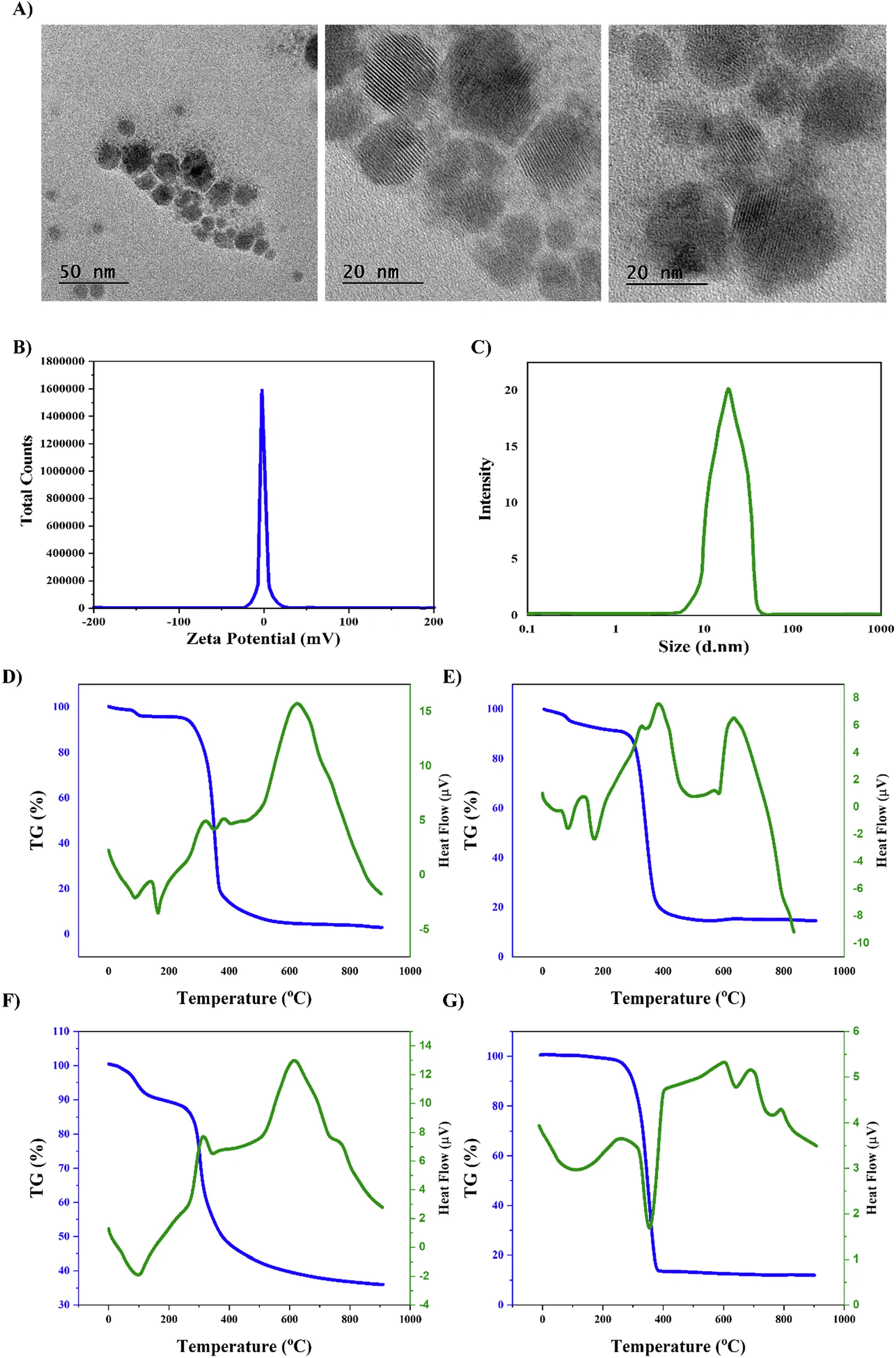
**A** TEM images of Rap@CS/PLGA NPs at 20 and 50 nm scale shows uniform, spherical nanoparticles and distinct lattice fringes, confirming structural integrity; **B** Zeta potential measurements show a stable surface charge, suggesting good colloidal stability of the nanoparticle suspension; **C** Dynamic light scattering data indicate a uniform particle size distribution with average diameters in the nanometer range. TGA curves of Rap@CS/PLGA NPs demonstrate variations in thermal degradation patterns, reflecting enhanced stability in the loaded nanoparticles: **D** CS/PLGA NPs; **E** PLGA NPs; **F** CS; and **G** PLGA

Transmission electron microscopy (TEM) images revealed that both Rap@PLGA and Rap@CS/PLGA nanoparticles were spherical, smooth-surfaced, and monodispersed (Fig. 2A). The encapsulation efficiency (%EE) of Rap was found to be 62.33 ± 0.87% for Rap@PLGA NPs and 60.30 ± 1.20% for Rap@CS/PLGA NPs, with no significant difference.

The mean diameters of CS/PLGA nanoparticles and Rap@CS/PLGA nanoparticles were 208.6 ± 9.07 nm and 265.9 ± 6.30 nm, respectively, indicating a slight increase in particle size following drug loading and chitosan coating. The corresponding polydispersity index (PDI) values were 0.211 ± 0.05 and 0.215 ± 0.12, confirming a uniform and narrowly distributed nanoparticle population (Table 2).

**Table 2:** Physicochemical characterization of Rap@CS/PLGA NPs

Parameter	CS/PLGA NPs	Rap@CS/PLGA NPs
Particle size (nm)	208.6 ± 9.07	265.9 ± 6.30
PDI	0.211 ± 0.0	0.215 ± 0.12
Zeta potential (mV)	−2.70 ± 3.50	+24.83 ± 2.12
Encapsulation efficiency (%EE)	–	60.30 ± 1.20

Data are expressed as mean ± SD (*n* = 3). The table presents particle size, polydispersity index (PDI), zeta potential, and encapsulation efficiency (%EE) of the formulations. Chitosan coating increased nanoparticle size and shifted the zeta potential from negative to positive, confirming successful surface modification. Both formulations exhibited narrow size distribution and high encapsulation efficiency, with no significant difference observed between them (*p* > 0.05)

Zeta potential measurements further confirmed surface modification: Rap@PLGA NPs displayed a surface charge of −2.70 ± 3.50 mV, confirming that (–) zeta potential attributed to terminal carboxyl groups on PLGA and possible PVA residues. Upon chitosan coating, Rap@CS/PLGA NPs exhibited a markedly positive zeta potential (+24.83 ± 2.12 mV), indicating successful adsorption of positively charged chitosan onto the nanoparticle surface (Fig. 2B, C).

In accordance with the thermal decomposition profile of pure PLGA, thermogravimetric analysis (TGA) of PLGA nanoparticles revealed a distinctive two-step degradation pattern, with significant weight loss events occurring between 350 °C and 630 °C. As shown in Fig. 2(D–G), chitosan and chitosan-coated nanoparticles (CS/PLGA NPs) exhibited a significant degradation peak at approximately 613 °C, indicating the presence of chitosan on the nanoparticle surface. Additionally, all samples exhibited an endothermic event at approximately 170°C, which was attributed to the cryoprotectant mannitol. Additionally, TGA analysis revealed no appreciable mass loss close to acetone’s boiling point (56 °C), indicating total solvent removal during the manufacturing process.

The physical mixture of Rap with polymers exhibited clear melting peaks at approximately 185.7 °C and 195.6 °C, which are typical of crystalline Rap, as well as peaks from PLGA and chitosan, as determined by differential scanning calorimetry (DSC). This suggests that there is no chemical interaction between the drug and excipients (Data not shown). The thermograms of the blank and Rap-loaded nanoparticles, on the other hand, revealed profiles that resembled those of the polymer matrix by itself. There were no discernible Rap peaks, indicating that the drug had been successfully encapsulated and that the drug’s crystallinity had probably decreased during entrapment. Mannitol was consistently identified by a thermal transition at about 166 °C.

The hydrophilic characteristic of Rap@CS/PLGA NPs was demonstrated by contact angles that were less than 90°. The CS/PLGA NPs displayed a contact angle of 74.1°, which may have been caused by PVA residues attached to the surface that persisted after several washes, a common occurrence when acetone is utilized as the organic solvent. The contact angle of CS/PLGA nanoparticles increased marginally to 77.6°, most likely due to the relative hydrophobicity of chitosan compared to PVA.

**Fig. 3:**
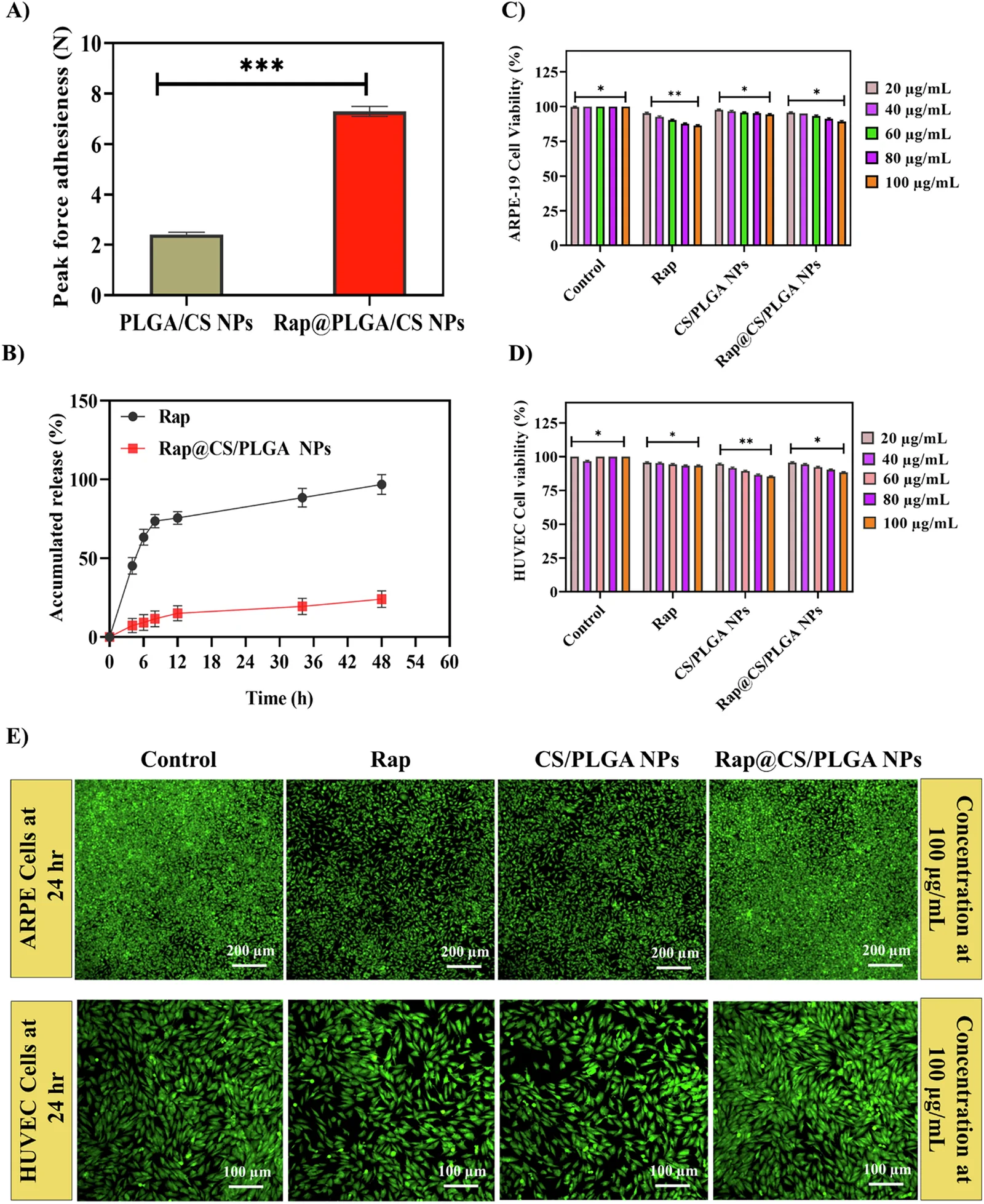
**A** Bioadhesion study showing higher detachment force for Rap@CS/PLGA NPs compared to CS/PLGA NPs on goat sclera; **B** Drug release profile of Rapamycin from Rap@PLGA/CS nanoparticles under simulated physiological conditions, showing a sustained release pattern compared to the free drug. In vitro cytocompatibility of Rap@CS/PLGA nanoparticles. **C**–**E** Cell viability of ARPE-19 and HUVEC cells after 24 h exposure to different concentrations (10–200 μg/mL) of free Rap, blank CS/PLGA NPs, and Rap@CS/PLGA NPs, measured by MTT assay. Data are presented as mean ± SD (*n* = 3). No significant cytotoxicity was observed up to 100 μg/mL, confirming biocompatibility of the nanocarrier system. *P < 0.05; **P < 0.01; ***P < 0.001 compared to the control group

The adhesive strength of CS/PLGA NPs was recorded as 1.4 ± 0.42 N, whereas the Rap@CS/PLGA NPs demonstrated a significantly stronger adhesion force of 6.93 ± 2.27 N, as illustrated in Fig. 3A. The moderate mucoadhesion seen with CS/PLGA NPs may result from traces of polyvinyl alcohol (PVA) remaining on the surface, as also indicated by contact angle measurements. Subconjunctival drug delivery is often limited by rapid clearance and a short residence time; thus, strategies that extend drug retention and offer sustained release are vital for improving delivery to posterior ocular tissues and minimizing the need for repeat administrations. In this study, the enhanced mucoadhesive property of Rap@CS/PLGA NPs is likely due to the positive charge and adhesive nature of chitosan at neutral or slightly basic pH conditions, which are typically found in the eye.

In the stability study, the lyophilized Rap@PLGA and Rap@CS/PLGA nanoparticles were stable in particle size, PDI, and %EE while stored at 4 °C for six months, with no significant alterations (*p* > 0.05). Six months at 25 °C resulted in a slight increase in Rap@PLGA nanoparticle size, as indicated in Table 2. However, particle size was acceptable. Rap@CS/PLGA nanoparticles’ greater zeta potential and electrostatic repulsion reduced particle aggregation, resulting in improved size stability under both storage regimes. None of the formulations changed color, transparency, or sedimentation during storage, as observed by the naked eye.

### In vitro drug release study

Understanding the release kinetics of therapeutic agents from nanocarriers is essential for achieving sustained drug delivery with reduced systemic toxicity. As shown in Fig. 3B, the Rap suspension exhibited a rapid and nearly complete release profile, with cumulative release reaching approximately 98 ± 2.3% within 48 h. In contrast, Rap@CS/PLGA NPs exhibited a significantly slower and controlled release, with only ~8.6 ± 1.2% released in the first 6 h and a cumulative release of about 23.4 ± 2.7% over 48 h. This substantial reduction in the initial burst release and prolonged drug release profile may be attributed to the chitosan coating, which acts as a diffusion barrier, limiting the immediate desorption of surface-adsorbed drug molecules. The tight polymeric network formed by chitosan, especially in mildly acidic or neutral pH environments, likely contributes to the retardation of drug diffusion from the PLGA core. To further elucidate the release mechanism, the experimental data were fitted to various kinetic models, including zero-order, first-order, Higuchi, and Korsmeyer–Peppas models. Among these, the release profile of Rap@CS/PLGA NPs showed the highest correlation with the Higuchi and Korsmeyer–Peppas models (R² = 0.97 and 0.98, respectively), indicating a diffusion-controlled release mechanism. The release exponent (n) value (< 0.5) obtained from the Korsmeyer–Peppas model further confirmed that the drug release follows Fickian diffusion (Table 3). These findings demonstrate that CS-functionalized PLGA nanoparticles provide a controlled and sustained drug delivery system, which may reduce premature drug release and improve therapeutic efficacy in ocular applications.

**Table 3:** Drug release kinetics of Rap and Rap@CS/PLGA nanoparticles

Drug	Zero-order (R²) *Qt* = *Q₀* + *k₀t*	First-order (R²) *ln(Q₀−Qt) = lnQ₀* − *k₁t*	Higuchi (R²) *Qt* = *kH t*^1/2^	Korsmeyer–Peppas (*n*) *Qt/Q* ∞ = *kKP t*^*n*^
Rap (Free)	0.91 ± 0.02 (k₀ = 2.8 ± 0.2%/h)	0.96 ± 0.01 (k₁ = 0.085 ± 0.01 h⁻¹)	0.93 ± 0.02 (kH = 21 ± 2% h^0.5^)	0.48 ± 0.03 (*n* = 0.48 ± 0.03)
Rap@CS/PLGA NPs	0.92 ± 0.01 (k₀ = 0.52 ± 0.04%/h)	0.94 ± 0.02 (k₁ = 0.031 ± 0.005 h⁻¹)	0.97 ± 0.01 (kH = 3.15 ± 0.3% h^0.5^)	0.43 ± 0.02 (*n* = 0.43 ± 0.02)

Kinetic parameters and correlation coefficients (R²) obtained from zero-order, first-order, Higuchi, and Korsmeyer–Peppas models. Data are presented as mean ± SD (*n* = 3)

### In vitro cytocompatibility and cellular mechanistic studies

The effect of pure Rapamycin, unloaded CS/PLGA nanoparticles, and Rap@CS/PLGA nanoparticles on ARPE-19 and HUVEC cell viability was examined using the MTT assay over a concentration range of 20–100 µg/mL (Fig. 3C–E). All treatment groups maintained viability levels exceeding 85%, indicating minimal cytotoxicity. Notably, cells treated with Rap@CS/PLGA nanoparticles exhibited slightly higher viability than those exposed to free Rap, particularly at higher concentrations. This may be attributed to the nanoparticle formulation limiting sudden drug exposure and enabling gradual intracellular release. Fluorescence microscopy following acridine orange staining confirmed these results (Fig. 3D). Cells retained normal morphology with bright green fluorescence and no evidence of membrane damage, supporting the cytocompatibility of the nanoparticles in both retinal epithelial and vascular endothelial cells.

**Fig. 4:**
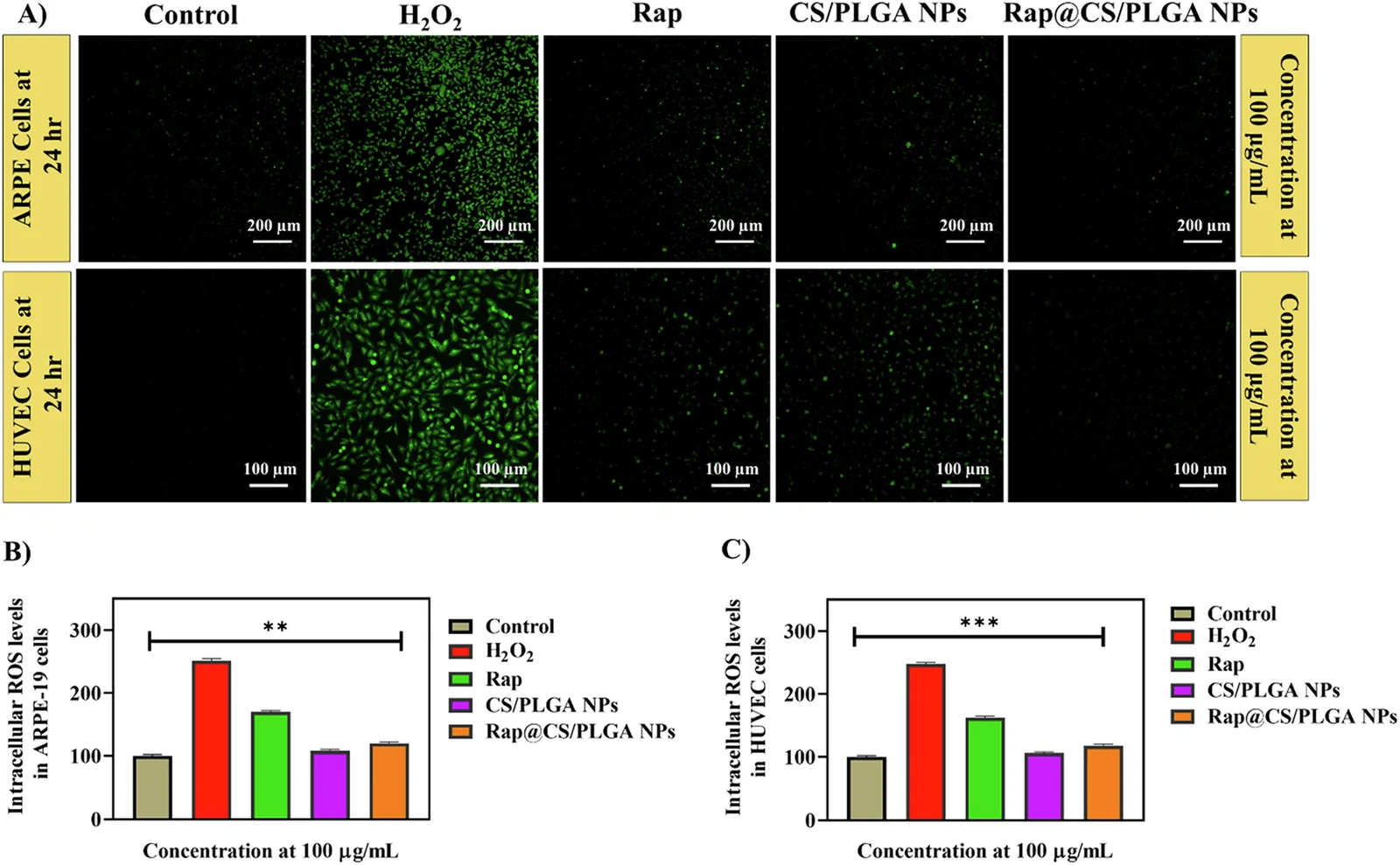
Inhibition of intracellular ROS generation by Rap@CS/PLGA NPs. **A** Representative fluorescence microscopy images of DCFDA-stained ARPE-19 and HUVEC cells showing green fluorescence intensity corresponding to reactive oxygen species (ROS) production. Cells treated with H₂O₂ exhibited strong fluorescence, while Rap@CS/PLGA NPs markedly suppressed ROS accumulation compared to free Rap and blank nanoparticles; **B, C** Quantitative analysis of fluorescence intensity confirms the antioxidant effect of Rap@CS/PLGA NPs. Values represent mean ± SD (*n* = 3, *p* < 0.01).All data are expressed as mean ± SD (*n* = 3 per group). *P < 0.05; **P < 0.01; ***P < 0.001 compared to the control group

Intracellular ROS generation, assessed using DCFDA staining, showed varying fluorescence intensities across treatment groups (Fig. 4A–C). H₂O₂-treated cells exhibited the strongest green fluorescence, validating the positive control. Free Rap induced moderate ROS production, whereas blank CS/PLGA nanoparticles produced minimal ROS comparable to untreated controls. Remarkably, Rap@CS/PLGA nanoparticles significantly reduced ROS levels in both ARPE-19 and HUVEC cells compared to free Rap (*p* < 0.001), suggesting effective modulation of intracellular oxidative stress through controlled drug release and chitosan coating.

**Fig. 5:**
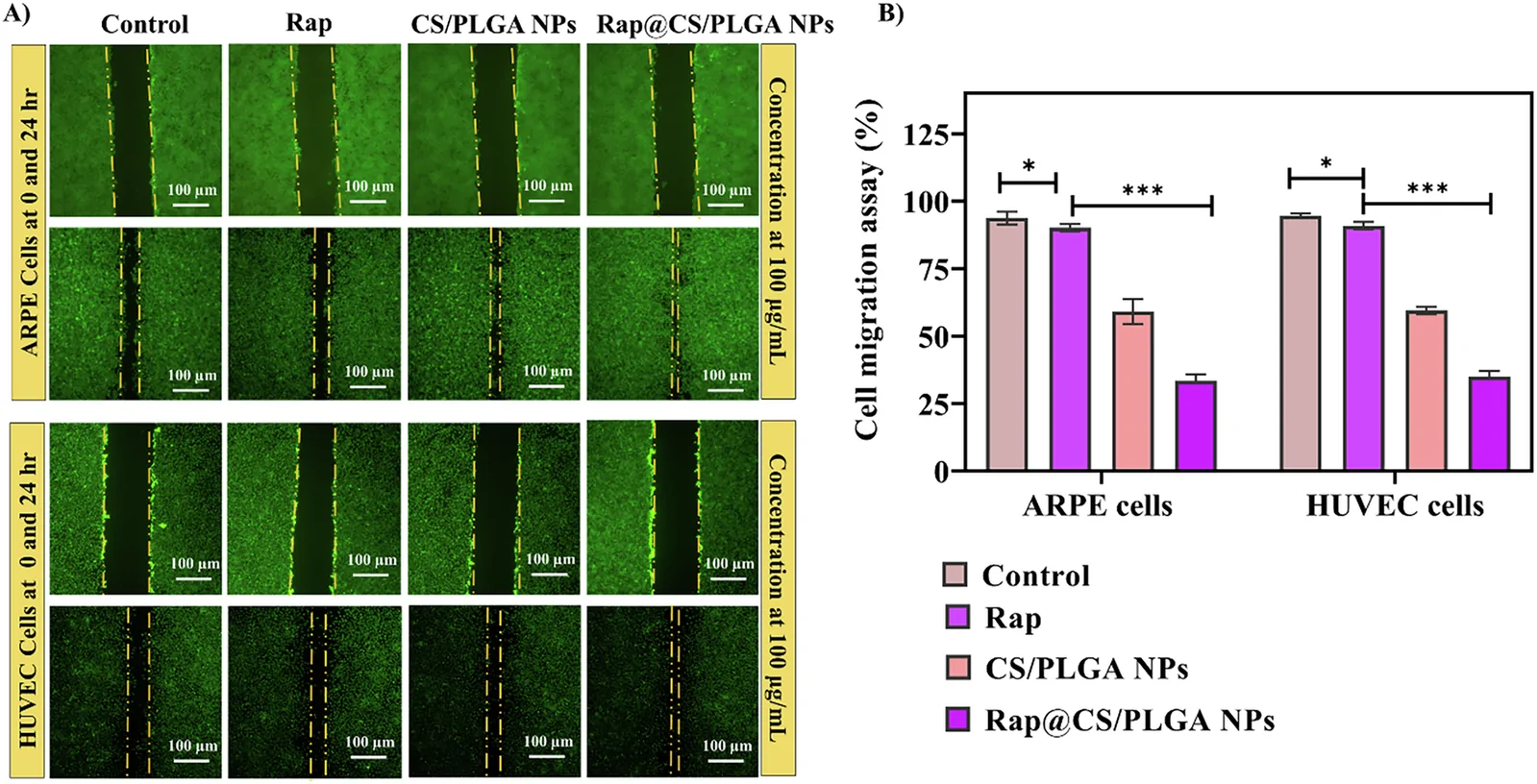
Cell migration (wound healing) assay of ARPE-19 and HUVEC cells. **A** Fluorescence micrographs showing wound closure at 0 h and 24 h for ARPE-19 and HUVEC cells treated with control, free Rap, CS/PLGA NPs, and Rap@CS/PLGA NPs (100 μg/mL). The yellow dashed lines mark wound margins. **B** Quantitative analysis (shows significant inhibition of cell migration in Rap@CS/PLGA NPs group compared to free Rap, indicating effective suppression of angiogenic cell motility. All data are expressed as mean ± SD (*n* = 3 per group). *P < 0.05; **P < 0.01; ***P < 0.001 compared to the control vs Rap group

The wound healing assay revealed distinct differences in cell migration (Fig. 5A, B). Untreated cells showed extensive gap closure after 24 h, while Rap@CS/PLGA nanoparticles markedly inhibited migration in both ARPE-19 and HUVEC cells. Free Rap moderately suppressed migration, and blank nanoparticles had a minor effect. The enhanced inhibition observed with Rap-loaded nanoparticles is likely due to sustained intracellular drug release, prolonging its biological activity and effectively regulating cell motility relevant to pathological migration and neovascularization.

**Fig. 6:**
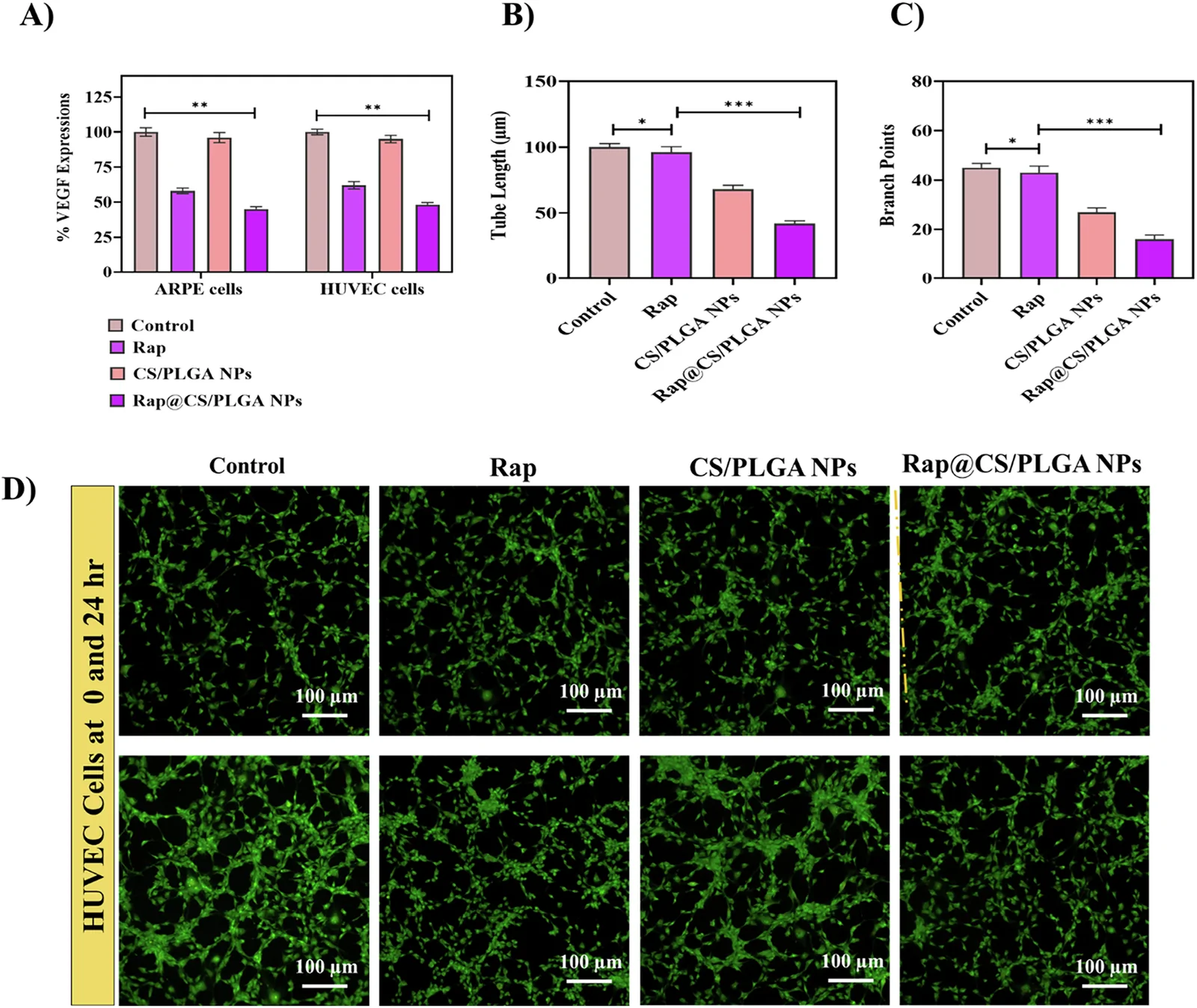
Anti-angiogenic effects of Rap@CS/PLGA nanoparticles. **A** Quantitative analysis of VEGF-A secretion in ARPE-19 and HUVEC cells after 24 h of treatment with control, free Rap, CS/PLGA NPs, and Rap@CS/PLGA NPs. Data (mean ± SD, *n* = 3) show that Rap@CS/PLGA NPs significantly reduced VEGF-A levels; **B**–**D** Representative tube formation assay images in HUVECs showing capillary-like network formation. Untreated cells formed extensive tubular structures, while Rap@CS/PLGA NPs markedly reduced total tube length and branch points compared to free Rap, confirming enhanced and sustained anti-angiogenic activity. All data are expressed as mean ± SD (*n* = 3 per group). *P < 0.05; **P < 0.01; ***P < 0.001 compared to the control vs Rap group

Quantification of VEGF-A secretion in cell culture supernatants indicated that Rap@CS/PLGA nanoparticles effectively downregulated VEGF-A in both ARPE-19 and HUVEC cells after 24 h of treatment (Fig. 6A). Free Rap produced a comparable reduction, whereas blank nanoparticles had no significant effect, confirming that the anti-angiogenic activity was driven by the active compound rather than the carrier system.

The functional anti-angiogenic potential of Rap@CS/PLGA nanoparticles was evaluated using a tube formation assay in HUVECs (Fig. 6B–D). Untreated HUVECs formed extensive capillary-like networks, while free Rap moderately disrupted tube formation, reducing total tube length and branch points. Rap@CS/PLGA nanoparticles exhibited a more pronounced inhibitory effect, with shorter tubes and fewer branch points, indicating sustained anti-angiogenic activity. Blank nanoparticles did not significantly alter tube formation compared to untreated controls. These results confirm that Rap@CS/PLGA NPs are cytocompatible yet effective at inhibiting endothelial angiogenesis.

### In vivo studies

#### Anti-angiogenic assay: in vivo

**Fig. 7:**
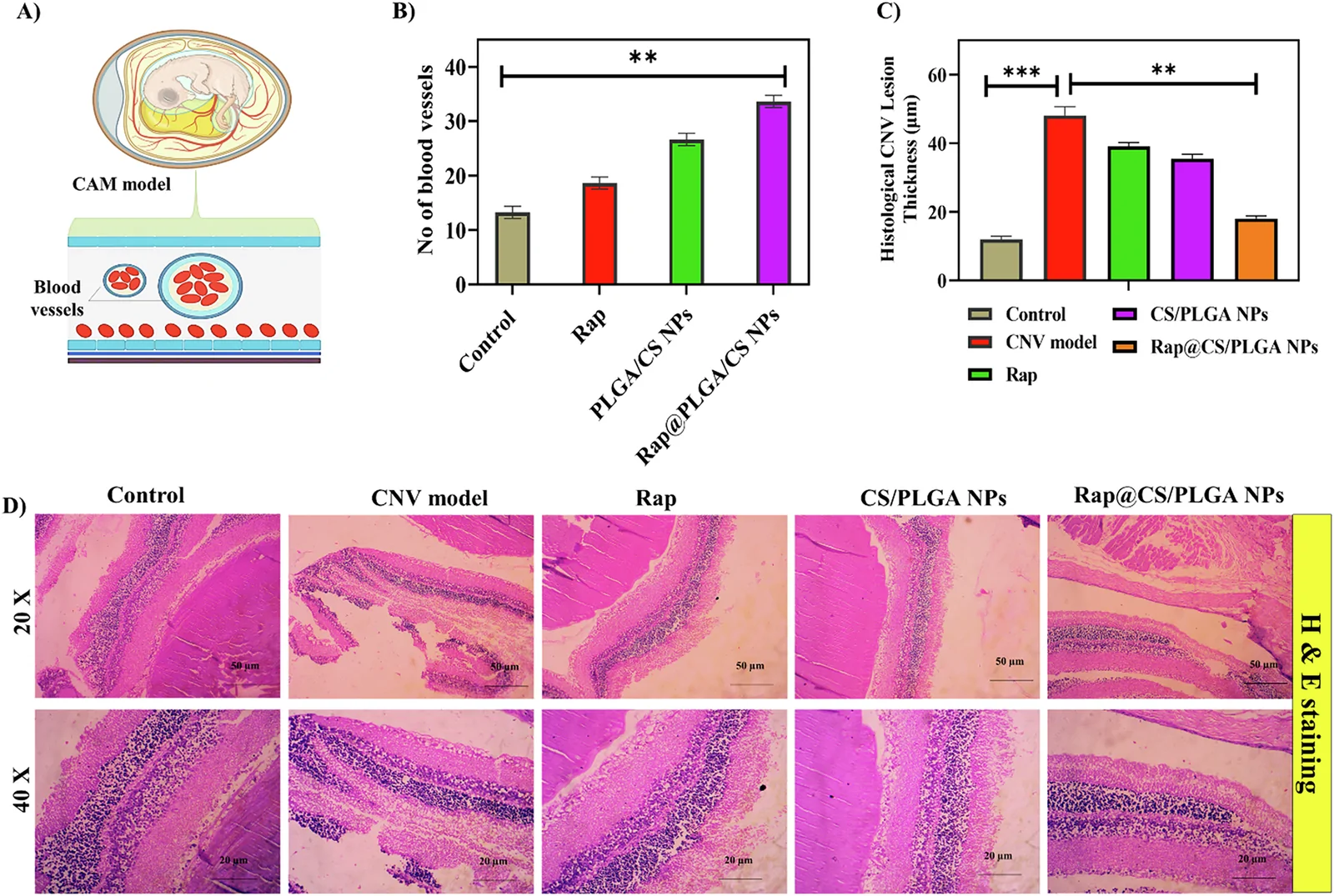
**A**, **B** In vivo CAM assay demonstrating anti-angiogenic effects. **C**, **D** Representative H&E-stained retinal sections and quantification of choroidal neovascular (CNV) lesion thickness across treatment groups. Eyes from PBS-treated CNV mice exhibited severe subretinal neovascularization and retinal disruption. Treatment with Rapamycin-loaded CS/PLGA nanoparticles significantly reduced CNV lesion thickness and preserved retinal architecture compared to PBS and unloaded nanoparticle groups. All data are expressed as mean ± SD (*n* = 3 per group). **P < 0.01; ***P < 0.001 compared to the control vs Rap group

The anti-angiogenic activity of the Rap@CS/PLGA NPs, each containing an equivalent Rap dose of 30 μg, was assessed by comparing them to normal saline. In the control group treated with normal saline, the number of blood vessels observed was 31 ± 3. In contrast, the Rap suspension treatment group exhibited a 26 ± 2% reduction in blood vessel count after 24 h. The CS/PLGA NPs group demonstrated a moderate reduction in blood vessel formation, with a count of 19 ± 2. However, the most pronounced decrease in blood vessel formation was observed in the Rap@CS/PLGA NPs group, where the blood vessel count dropped significantly to 13 ± 3. This reduction was statistically significant compared to both the saline-treated group (*p* < 0.0001) and the Rap suspension group (*p* < 0.01). Additionally, in the Rap@CS/PLGA NPs group, a more significant disruption of the capillary network was observed, resulting in a larger avascular zone compared to the saline-treated group (Fig. 7A, B). These findings demonstrate the enhanced anti-angiogenic effect of Rap@CS/PLGA NPs, suggesting their potential as an effective therapeutic option for treating wet age-related macular degeneration (wet-AMD).

#### Evaluation of Rap@CS/PLGA nanoparticles in a murine model of choroidal neovascularization

Histological analysis using hematoxylin and eosin (H&E) staining revealed marked pathological changes in the retinal and choroidal layers of CNV-induced eyes. In the PBS-treated CNV group, extensive subretinal neovascularization and disruption of retinal architecture were observed, including thickening of the choroid and loss of outer retinal layers. In contrast, mice treated with Rapamycin-loaded CS/PLGA nanoparticles exhibited a preserved retinal structure with significantly reduced neovascular lesion thickness. The unloaded nanoparticle group showed only modest improvement compared to PBS controls, indicating a drug-specific therapeutic effect (Fig. 7C, D).

**Fig. 8:**
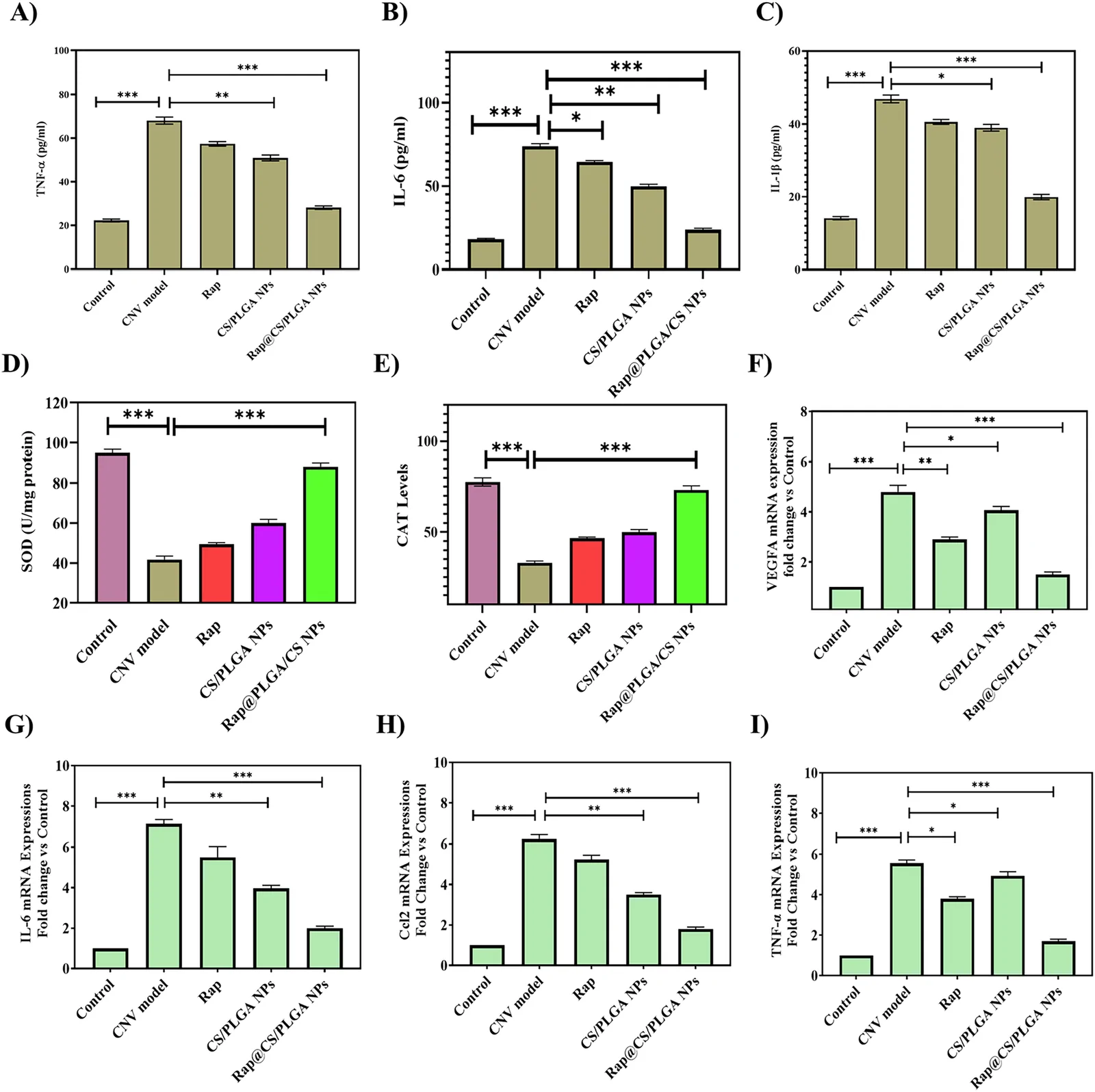
ELISA quantification of **A** TNF-α; **B** IL-6; **C** IL-1β protein levels in RPE-choroid lysates from different treatment groups on day 7 post-laser injury. PBS-treated CNV eyes showed elevated cytokine levels, indicating inflammation. Rap@CS/PLGA NPs significantly reduced cytokine levels compared to PBS and CS/PLGA groups, demonstrating potent anti-inflammatory effects. **D** Superoxide dismutase (SOD) and **E** catalase (CAT) activities in RPE-choroid homogenates following CNV induction and treatment. Both enzyme activities were significantly suppressed in the PBS-treated CNV group, indicative of oxidative stress. Rap@CS/PLGA NPs significantly restored antioxidant activity to levels comparable to sham controls, while unloaded nanoparticles had only moderate effects. Relative mRNA expression levels of **F** VEGFA; **G** IL-6; Ccl2**;** **H** Ccl2 and **I** TNF-α in RPE-choroid tissues. PBS-treated CNV eyes exhibited strong upregulation of angiogenic and inflammatory genes. Rap@CS/PLGA NPs significantly downregulated all four genes, while unloaded nanoparticles produced partial reductions in Ccl2 and IL-6 but not VEGFA or TNF-α. All data are expressed as mean ± SD (*n* = 3 per group). *P < 0.05; **P < 0.01; ***P < 0.001 compared to the control vs Rap group

Quantitative ELISA assays of RPE-choroid lysates demonstrated a significant upregulation of pro-inflammatory cytokines TNF-α, IL-6, and IL-1β in the PBS-treated CNV group compared to sham controls (*p* < 0.01). Treatment with Rapamycin-loaded CS/PLGA nanoparticles significantly reduced the expression of all three cytokines, TNF-α and IL-6 (*p* < 0.01); IL-1β (*p* < 0.05), restoring levels to near baseline. In contrast, the unloaded nanoparticle group produced only a partial reduction in cytokine levels, suggesting the anti-inflammatory efficacy was attributable primarily to Rapamycin (Fig. 8A–C).

To investigate whether Rap@CS/PLGA NPs mitigate oxidative stress associated with CNV, the key antioxidant enzymes like SOD and CAT were measured in RPE-choroid tissue homogenates on day 7 following laser injury (Fig. 8D, E). In PBS-treated CNV mice, both SOD and CAT activities were significantly reduced compared to healthy controls (SOD: *p* < 0.01; CAT: *p* < 0.01), indicating an oxidative imbalance resulting from neovascular and inflammatory insults. Treatment with Rap@CS/PLGA NPs led to a marked restoration of antioxidant defense. Specifically, SOD activity was significantly increased in the Rap@CS/PLGA group compared to the PBS group (*p* < 0.01), reaching levels comparable to non-lasered controls. Similarly, CAT activity was significantly elevated (*p* < 0.01), suggesting effective enzymatic neutralization of reactive oxygen species (ROS). In contrast, eyes treated with unloaded CS/PLGA NPs exhibited only modest increases in SOD and CAT levels, which did not reach statistical significance relative to the PBS group. These results indicate that Rap@CS/PLGA NPs not only inhibit inflammation and neovascularization but also restore redox homeostasis in CNV-damaged tissue, highlighting their multi-modal therapeutic potential.

Consistent with the cytokine protein levels, qPCR analysis revealed significantly elevated mRNA expression of VEGFA, CCL2, IL-6, and TNF-α in CNV eyes treated with PBS. Rapamycin-loaded nanoparticle treatment significantly suppressed the expression of these genes (*p* < 0.01), indicating effective downregulation of both angiogenic and inflammatory signaling pathways at the transcriptional level. The unloaded nanoparticle group showed moderate suppression of CCL2 and IL6, but did not significantly affect VEGFA or TNF-α, further underscoring the role of Rapamycin in modulating CNV-associated molecular responses (Fig. 8F–I).

## Discussion

PLGA-based nanocarriers and chitosan surface modification have been widely investigated for ocular drug delivery, with several studies demonstrating enhanced ocular retention and therapeutic efficacy of chitosan-coated PLGA systems for anti-inflammatory and hypotensive agents such as diclofenac sodium and dorzolamide [25, 26]. Comprehensive reviews have further established nano-carrier–based strategies as a mature and evolving approach in ophthalmic drug delivery [27]. In parallel, the anti-angiogenic potential of rapamycin in ocular neovascular disorders is well documented, including its inhibitory effects on retinal and choroidal neovascularization and VEGF expression via mTOR pathway suppression [28, 29]. Accordingly, the present study does not propose a fundamentally new nanocarrier platform, but rather focuses on functional refinement and biological validation of a chitosan-functionalized PLGA system for rapamycin delivery in neovascular age-related macular degeneration (wet AMD). Compared with previously reported rapamycin nanoformulations, including chitosan/PLA nanoparticles designed primarily for antiproliferative delivery [30], as well as lipid-based carriers, polymeric micelles, and surfactant-modified PLGA systems, the formulation developed here exhibits distinct functional advantages. Chitosan functionalization imparted a positively charged and hydrophilic surface, consistent with prior ocular nanoparticle studies [31], resulting in enhanced scleral mucoadhesion and suggesting improved local ocular residence. In addition, the system demonstrated controlled drug release with attenuation of the initial burst phase, a feature relevant for minimizing acute retinal exposure and reducing toxicity risks associated with free rapamycin. Importantly, this study extends beyond formulation-level optimization by providing multilevel biological validation that has not been comprehensively reported for earlier rapamycin-loaded polymeric systems. Therapeutic efficacy was consistently demonstrated across in vitro assays, chorioallantoic membrane angiogenesis screening, and a laser-induced choroidal neovascularization mouse model. Treatment resulted in marked suppression of pathological angiogenesis, accompanied by downregulation of pro-inflammatory cytokines (TNF-α, IL-6, IL-1β) and restoration of endogenous antioxidant activity. Moreover, direct comparison with free rapamycin confirmed that nanoparticle-mediated delivery produced superior anti-angiogenic and anti-inflammatory outcomes at equivalent doses, supporting a functional rather than purely compositional improvement. Collectively, these findings position the present system as an incremental yet biologically substantive advancement, offering a translationally relevant optimization of established nanocarrier strategies for wet AMD therapy.

The successful synthesis of chitosan-coated PLGA nanoparticles (CS/PLGA NPs) was confirmed using various physicochemical techniques. The electrostatic attraction between anionic PLGA and cationic chitosan enabled the stable adsorption of chitosan onto the nanoparticle surface, thus improving stability through steric and electrostatic repulsion, in accordance with previous findings [21, 32, 33]. Furthermore, encapsulation of Rap in PLGA-based NPs has shown the capability of sustained drug release and improved intracellular delivery, leading to superior inhibition of the mTOR pathway compared with Rap drug [34]. Numerous reports indicated that PLGA NPs enhance drug stability, bioavailability, and therapeutic efficacy. Comparative studies with other nanocarriers, like lipid nanocapsules, underscore the advantages of PLGA systems in controlled release and gene-regulatory effects [35–37]. These results collectively support the rational design of CS/PLGA NPs as an effective platform for Rap delivery with enhanced pharmacological performance. FTIR spectra exhibited diminished Rap peaks in the NPs formulation, indicating effective encapsulation and possible drug-polymer interactions [38, 39]. XRD patterns showed that Rap changed from a crystalline to an amorphous/molecularly dispersed state, which is beneficial for enhancing solubility and bioavailability [40]. TEM analysis showed that NPs were spherical and smooth, which is necessary for reproducible drug delivery. The encapsulation efficiency was similar for both uncoated and CS functionalized NPs, indicating that surface modification did not adversely impact drug loading, in agreement with earlier reports [41]. The minor augmentation in particle size post-chitosan coating and Rap loading is presumably attributable to hydrogen bonding and surface adsorption. A restricted size distribution is important for predictable in vivo performance, and low PDI values show this. The zeta potential changed from negative to highly positive after coating with chitosan shows that chitosan’s amine groups were able to change the surface [42]. DSC analysis corroborated with encapsulation results, evidenced by the disappearance of Rap in NPs formulations. The lack of additional thermal transitions indicates that no chemical interaction transpired between Rap and the excipients [15, 41]. GC analysis and TGA confirmed that complete solvent removal, which is in line with safety requirements [43, 44]. The contact angle increasing to 77.6° for CS/PLGA NPs means that the surface is more hydrophobic because chitosan is less hydrophilic than residual PVA [45, 46]. This surface property improved mucoadhesion due to the positively charged chitosan, which interacted effectively with negatively charged ocular tissues, potentially increasing residence time for ocular drug delivery [21]. The contribution of chitosan to the enhanced performance of Rap@CS/PLGA nanoparticles is interpreted cautiously. Although chitosan is widely reported to promote mucoadhesion and cellular interaction in ocular delivery systems [25], direct quantitative evaluation of permeability, cellular uptake, or posterior segment transport was not performed in the present study, and mechanistic effects are therefore inferred rather than demonstrated. In this study, the proposed role of chitosan is supported by indirect experimental evidence, including a positive surface charge, increased scleral mucoadhesive force, and improved biological efficacy at equivalent rapamycin doses. Accordingly, claims of enhanced posterior segment delivery remain hypothetical, and quantitative transport, uptake, and biodistribution studies are identified as essential future investigations. Long-term stability studies showed that both lyophilized CS/PLGA NPs and Rap@CS/PLGA NPs remained stable in size, PDI, and drug entrapment efficiency at 4 °C. Rap@CS/PLGA NPs showed superior stability at 25 °C, likely due to greater zeta potential preventing aggregation. In vitro release profiles showed that Rap@CS/PLGA NPs could release their contents over time (~23.4% in 48 h), but free Rap could release its contents quickly (~98% in 48 h). The chitosan layer likely formed a semipermeable barrier, limiting drug diffusion, particularly at neutral pH. This controlled release aligns with previous studies, where chitosan’s low solubility above pH 6.3 enabled the formation of a hydrogel-like barrier [47, 48]. The 72 h in vitro release study was designed to assess initial burst suppression and early sustained release, which are critical for intravitreal safety and early therapeutic control, and to enable direct comparison with free rapamycin. While these results demonstrate moderated early release from Rap@CS/PLGA nanoparticles, extended release profiling and long-term intravitreal pharmacokinetic studies are required to fully evaluate injection-frequency reduction and remain a focus of future work. Cytocompatibility testing in both ARPE-19 and HUVEC cells revealed >85% cell viability across all formulations, with Rap@CS/PLGA NPs exhibiting superior tolerance compared to free Rap, particularly at higher concentrations. Acridine orange staining confirmed the maintenance of cellular morphology and membrane integrity, indicating that the regulated intracellular release facilitated by the NPs matrix mitigates sudden drug exposure and potential cytotoxicity. These results are consistent with prior studies indicating that chitosan can exhibit dose-dependent cytotoxicity above certain thresholds; however, our formulation remained well within safe limits [49]. Assessment of oxidative stress using DCFH-DA staining revealed that free Rap and H₂O₂ induced substantial ROS generation, whereas Rap@CS/PLGA nanoparticles significantly suppressed intracellular ROS levels in both cell types. This shows that the nanoparticle method can reduce oxidative stress by releasing drugs over time and that chitosan itself has antioxidant capabilities [50, 51]. Functionally, Rap@CS/PLGA nanoparticles also effectively inhibited VEGF-A secretion in ARPE-19 and HUVEC cells and markedly suppressed tube formation in HUVECs, demonstrating potent anti-angiogenic activity. The combination of high cytocompatibility, reduced oxidative stress, and anti-angiogenic potential underscores the therapeutic promise of Rap@CS/PLGA nanoparticles for retinal disorders characterized by pathological angiogenesis and oxidative injury. In vivo, anti-angiogenic efficacy was evaluated using both the CAM assay and a laser-induced murine model of choroidal neovascularization (CNV). The CAM assay was employed as a preliminary angiogenesis screening model and does not replicate the ocular microenvironment; therefore, disease-relevant in vivo validation in the present study is primarily supported by the laser-induced CNV mouse model, which more closely reflects the pathological features of wet age-related macular degeneration. The chorioallantoic membrane (CAM) assay was used solely as a rapid in vivo screening model to assess the general anti-angiogenic activity of Rap@CS/PLGA nanoparticles. Although well established for angiogenesis evaluation [52], the CAM model does not reproduce key features of the retinal or choroidal microenvironment, including retinal architecture, blood–retinal barriers, and ocular pharmacokinetics. Accordingly, CAM findings were interpreted as preliminary and not as direct evidence of therapeutic efficacy in wet age-related macular degeneration. In contrast, disease relevance in the present study is primarily supported by the laser-induced choroidal neovascularization (CNV) mouse model, which more closely reflects pathological choroidal angiogenesis characteristic of wet AMD [53]. Rap@CS/PLGA NPs significantly decreased neovascularization (13 ± 3 vessels), compared to saline (31 ± 3) and free Rap (26 ± 2), and also enlarged avascular zones in the CAM assay. The CNV model showed the same effects, with Rap@CS/PLGA treatment well maintaining retinal structure and reducing lesion thickness. Moreover, this formulation markedly downregulated the inflammatory cytokines TNF-α, IL-6, and IL‑1β in ocular tissues. These cytokines are key mediators of inflammatory angiogenesis in AMD, and their inhibition further supports Rap immunomodulatory function [54, 55]. Importantly, Rap@CS/PLGA treatment significantly reinstated antioxidant enzyme levels (SOD and CAT) in CNV eyes, rectifying the oxidative imbalance linked to neovascular AMD [56–58]. Gene expression analysis validated the downregulation of VEGFA, CCL2, IL6, and TNF-α, underscoring the formulation’s dual anti-angiogenic and anti-inflammatory properties. These results are consistent with previous findings on Rap ability to inhibit VEGF and mitigate NF-κB–driven inflammation [59, 60]. Together, this study emphasized the therapeutic potential of Rap@CS/PLGA as a multifunctional, sustained-release intravitreal solution for the management of wet AMD. The formulation targets vital pathological aspects: angiogenesis, inflammation, and oxidative stress, offering advantages over conventional anti-VEGF therapies. In current clinical practice, anti-VEGF agents such as bevacizumab and ranibizumab are widely used but require frequent intravitreal injections and are associated with potential complications. In contrast, Rap@CS/PLGA nanoparticles provide a broader mechanism by targeting mTOR-mediated angiogenesis, inflammation, and oxidative stress, along with sustained drug release, which may reduce dosing frequency and improve therapeutic outcomes [61]. Subsequent research must concentrate on the long-term safety of the retina, the regulation of autophagy, and the Nrf2 antioxidant pathway to further substantiate this delivery platform. While the present findings demonstrate robust anti-angiogenic, anti-inflammatory, and antioxidant efficacy in established preclinical models, this study should be regarded as a proof-of-concept evaluation rather than definitive translational validation. Detailed ocular pharmacokinetics, retinal biodistribution, and long-term intravitreal retention were beyond the scope of the current work and, therefore, not assessed. These parameters are critical for determining dosing strategies, retinal exposure, and clinical translatability, and should be addressed in future studies to fully establish the therapeutic potential of Rap@CS/PLGA nanoparticles.

## Conclusion

This study highlights the potential of Rap@CS/PLGA NPs as a promising strategy for ocular drug delivery in the treatment of wet-AMD. The nanoparticles exhibited high encapsulation efficiency and a controlled, sustained drug release profile, vital features for reducing dosing frequency and enhancing patient adherence. In vitro studies confirmed minimal cytotoxicity and effective mitigation of oxidative stress in both retinal epithelial (ARPE-19) and vascular endothelial (HUVEC) cells. Moreover, Rap@CS/PLGA NPs effectively suppressed VEGF secretion, inhibited cell migration, and disrupted HUVEC tube formation, which indicates potent anti-angiogenic activity. In vivo administration of Rap@CS/PLGA NPs in a laser-induced CNV mouse model leads to marked suppression of pathological neovascularization, decreased inflammatory cytokines, restoration of antioxidant levels, and modulation of angiogenesis-related gene expression, which also confirms the dual therapeutic action of the Rap@CS/PLGA NPs. Overall, these findings demonstrated that Rap@CS/PLGA NPs are a safe, effective, and site-specific delivery platform capable of simultaneously mitigating oxidative stress and pathological angiogenesis, offering their potential as a next-generation therapy for neovascular ocular disorders. Accordingly, the present study establishes preclinical proof-of-concept efficacy, with further pharmacokinetic, biodistribution, large-animal, and long-term release studies required to support clinical translation.

## Data Availability

The data is accessible upon request. This released article has all the data that was collected or analyzed during the study.
